# The Efficacy of Self-Management Strategies for Females with Endometriosis: a Systematic Review

**DOI:** 10.1007/s43032-022-00952-9

**Published:** 2022-04-29

**Authors:** Amelia K. Mardon, Hayley B. Leake, Cathy Hayles, Michael L. Henry, Patricia B. Neumann, G. Lorimer Moseley, K. Jane Chalmers

**Affiliations:** 1 IIMPACT in Health, University of South Australia, Adelaide, SA Australia; 2 Centre for IMPACT, Neuroscience Research Australia, Sydney, NSW Australia; 3 University of South Australia, Adelaide, SA Australia; 4 Western Sydney University, Campbelltown, NSW Australia

**Keywords:** Endometriosis, Self-management, Systematic review, Diet, Supplements, Exercise

## Abstract

Self-management is critical for the care of endometriosis. Females with endometriosis frequently use self-management strategies to manage associated symptoms; however, the efficacy of such strategies is unknown. The aim of this review was to systematically appraise the evidence concerning efficacy of self-management strategies for endometriosis symptoms. Electronic databases, including Medline, Embase, Emcare, Web of Science Core Collection, Scopus, and the Cochrane Central Register of Controlled Trials, were searched from inception to March 2021. We included peer-reviewed experimental studies published in English evaluating the efficacy of self-management strategies in human females laparoscopically diagnosed with endometriosis. Studies underwent screening, data extraction, and risk of bias appraisal (randomised studies: Risk of Bias 2 tool; non-randomised studies: Risk Of Bias In Non-randomized Studies – of Interventions tool). Of the fifteen studies included, 10 evaluated dietary supplements, three evaluated dietary modifications, one evaluated over-the-counter medication, and one evaluated exercise. Most studies had a high-critical risk of bias. Many self-management strategies were not more effective at reducing endometriosis symptoms compared to placebo or hormonal therapies. Where studies suggest efficacy for self-management strategies, no recommendations can be made due to the poor quality and heterogeneity of evidence. High-quality empirical evidence is required to investigate the efficacy of self-management strategies for females with endometriosis.

**Supplementary Information:**

The online version contains supplementary material available at 10.1007/s43032-022-00952-9.

## Introduction

Endometriosis is a chronic inflammatory condition, characterised by the presence of endometrial-like tissue outside the uterine cavity, including the pelvic peritoneum, rectovaginal septum, and ovaries [1, 2]. An estimated 6–10% of reproductive-aged females are diagnosed with endometriosis [3, 4]. Endometriosis considerably impacts a person’s biological, psychological, and social wellbeing, with symptoms including pelvic pain, fatigue, and stress [5]. Endometriosis is associated with decreased quality of life, sub-fertility, and limited daily activities, such as attending work and school [6, 7]. Females with endometriosis often experience symptoms for years before receiving a diagnosis [8–10]; meanwhile, they consult many health professionals and trial various interventions to manage symptoms [11].

Interventions for endometriosis-associated symptoms result in suboptimal patient outcomes. Laparoscopic removal of endometriosis is the preferred treatment method, but is often associated with unchanged or worsening pain, and high rates of repeat surgery [12, 13]. Pharmaceutical interventions are common, including hormonal therapies and analgesics, but limited efficacy and bothersome side effects often lead to serial medication trials [14] and polydrug use [15]. Females with endometriosis frequently seek out other self-management methods to reduce symptoms and improve quality of life [16].

Self-management is a critical component of management of chronic conditions, including heart disease, asthma, low back pain, and osteoarthritis [17–21]. Evidence-based healthcare support focusing on self-care is endorsed internationally [22]. In the absence of a ‘gold standard’ definition for self-management [18], the current review refers to self-management as the ability of an individual to manage physical and psychosocial symptoms, treatments, and lifestyle changes associated with living with a chronic condition. We define self-management strategies as physical or psychological interventions (including lifestyle changes) that an individual can perform or administer themselves, specifically for the management of endometriosis symptoms [16].

Females with endometriosis report using self-management strategies, most commonly heat, rest, and meditation [16], and their use seems to be associated with increased quality of life [23]. A recent systematic review identified self-care activities and complementary therapies as important components of endometriosis self-management [24], but did not evaluate the efficacy of those strategies. We aimed to fill this critical gap by systematically reviewing the evidence concerning efficacy of self-management strategies for females with endometriosis.

## Methods

This review is reported in alignment with the Preferred Reporting Items for Systematic Review and Meta-Analysis (PRISMA) [25] and was prospectively registered on Open Science Framework (https://osf.io/gvepq/) and PROSPERO (CRD42021243107) on 16 March 2021.

### Literature Search

A search strategy for eligible studies was developed and piloted using medical subject headings and keywords, including ‘endometriosis’, ‘self-management’, and ‘self-care’ (see Supplementary File 1). Electronic databases were searched from inception to 24 March 2021, including: Medline, Embase, EmCare, Web of Science Core Collection, Scopus, and the Cochrane Central Register of Controlled Trials. We performed an internet search of Google Scholar using similar keywords; we reviewed the first 100 references on the premise that the most relevant studies would appear first. Caches, cookies, and search history were cleared prior to undertaking the internet search. Websites of relevant organisations, reference lists of relevant reviews, clinical guidelines (see Supplementary File 1), and the reference lists of the included studies were manually searched to identify potentially relevant studies.

### Eligibility Criteria

We included studies that evaluated the efficacy and/or effectiveness of self-management strategies in females with endometriosis. For inclusion, studies had to: 1) recruit human females (of any age) diagnosed with endometriosis via laparoscopy or histological confirmation; 2) evaluate the efficacy and/or effectiveness of self-management strategies (as defined previously), on self-report endometriosis-associated symptoms; 3) be peer-reviewed of any experimental study design (e.g. randomised controlled trials, non-randomised controlled trials, cohort studies); and 4) be reported in English. We excluded studies that: 1) were interventions requiring administration by another individual (e.g. surgery, acupuncture); 2) included secondary data (e.g. reviews, commentaries); 3) were feasibility, animal, or in vitro studies.

### Selection Process

Studies identified by electronic databases were exported to Endnote (version X9.2, Clarivate, Philadelphia, USA) and then uploaded to Covidence (Veritas Health Innovation, Melbourne, Australia) where duplicates were removed. The review team (AM, CH, and MH) independently screened title and abstracts in duplicate (from AM, CH, and MH). The full texts of the remaining records were then independently screened by two reviewers (AM and MH) to confirm inclusion, with disagreements resolved by discussion. Relevant studies identified by the manual internet search and reference lists were also screened independently in duplicate. Where further information was required to confirm eligibility, we contacted corresponding authors up to two times within a four-week period. Data from authors that did not respond within the four-week period were deemed irretrievable and not included in this review.

### Data Extraction

Data were extracted from eligible studies independently by two reviewers (AM and CH) using a customised and pilot-tested data extraction form. Discrepancies or disagreements between reviewers were resolved by discussion or, if necessary, in consultation with a third reviewer (KJC). We extracted the following data: study and participant characteristics, and information about the intervention/s and control/s. The primary outcomes were pain-related endometriosis symptoms (e.g. dysmenorrhoea) assessed via self-report scales (e.g. visual analogue scale). Where available, we extracted secondary self-report outcome data, including depression, anxiety and/or stress scores, quality of life assessment, use of analgesic medication, and adverse events. Where data were provided graphically, data points were extracted independently by two reviewers (AM, CH) using Webplotdigitizer [26]. For these data points, the percentage difference between the two reviewers’ extracted data was calculated, and those that differed by > 10% required reconciliation. For the data points that differed by < 10%, the mean of the two data values was calculated and used for effect size calculations. Outcome data reported and collected across multiple time points were classified as baseline, end of intervention, short term (1–3 months post-intervention), intermediate (4–12 months post-intervention), and long term (over 12 months post intervention). Where insufficient data were reported within studies, corresponding authors were contacted two times via email, two weeks apart, requesting access. Data not retrieved within four weeks of the initial email were considered irretrievable.

### Risk of Bias Assessment

Two independent reviewers (AM, CH, or MH) appraised study-level risk of bias using one of two appraisal tools: the Cochrane Risk of Bias 2 (RoB2) [27] for randomised trials or the Risk Of Bias In Non-Randomized Studies – of Interventions (ROBINS-I) [28] for non-randomised studies. The RoB2 consists of five domains (the randomisation process, deviations from the intended interventions, missing outcome data, measurement of the outcome, selection of the reported result) whilst the ROBINS-I consists of seven domains (confounding, selection of participants into the study, classification of interventions, deviations from intended interventions, missing data, measurement of outcomes, and selection of the reported result). Responses to signalling questions within each domain answered included either yes, probably yes, probably no, no, or no information. The context of the signalling question determined whether the answers were high or low risk of bias. Individual domains and overall risk of bias were classified as either low, high, or unclear. The risk of bias judgement at the domain and overall level were determined using the algorithms provided with the risk of bias tools. Disagreements between appraisals of risk of bias were resolved by discussion with a third reviewer (KJC).

The quality and certainty of the cumulative evidence were not appraised using the Grading of Recommendations Assessment, Development and Evaluation (GRADE) framework. This was a deviation from our original protocol because the heterogeneity between studies, including in study design, participant populations, and interventions used, and exclusion of meta-analysis, meant that a GRADE recommendation would not be possible.

### Data Synthesis and Analysis

Included studies were grouped according to the type of self-management strategy assessed. Where appropriate data were available, effect sizes were calculated for continuous measures of pain outcomes using Review Manager software (version 5.4.1, Cochrane, United Kingdom). Effect sizes were presented in bar charts as standardised mean differences (SMD) and 95% confidence intervals (CI) due to heterogeneity of assessment tools. Narrative synthesis compiled outcome data not appropriate for effect size calculations. Clinical and methodological heterogeneity between studies, and statistical heterogeneity meant that we chose not to undertake sensitivity analyses.

## Results

### Study Selection and Characteristics of Included Studies

**Fig. 1:**
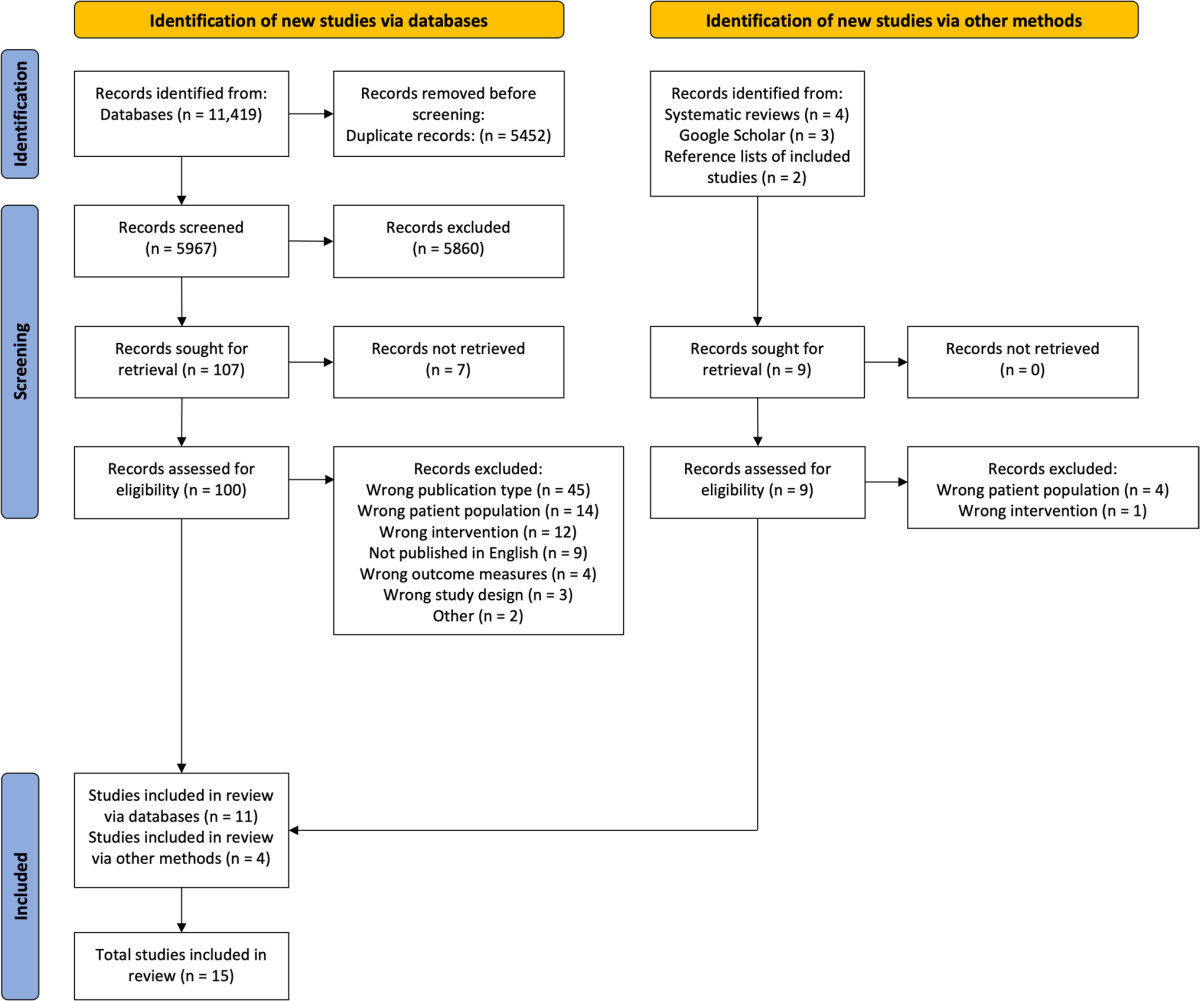
PRISMA flowchart of included studies. Legend: Preferred Reporting Items for Systematic Review and Meta-Analysis, PRISMA

Figure 1 shows the PRISMA flow diagram (see Supplementary File 2 for excluded studies). Fifteen studies (*n* = 1093; 660 allocated to intervention; 759 to comparator) were eligible for this review (Table 1), including eight randomised controlled trials (RCT) [29–38], two observational case series [39, 40], one non-randomised comparative trial [41], one cross-over trial [42], and one pre-post intervention trial [43].

**Table 1:** Characteristics of included studies

Citation	Study design	Location	Population	Intervention; number of participants	Comparator; number of participants	Co-interventions	Intervention duration/ follow-up	Outcome measures
*Dietary supplements*								
(Almassinokiani et al*.*, 2016)	RCT	Iran	Mean age 30 ± 5; Baseline VAS score (10 cm) > 3; Endometriosis stage mild-severe	Vitamin D supplement: One oral capsule of 50 000 IU Vitamin D3 per week; *n* = 19	Placebo: One oral capsule per week; *n* = 20	All participants: conservative diagnostic surgery (8 weeks pre-intervention)	12 weeks/post-intervention (EOI)	10 cm VAS Dysmenorrhoea, non-menstrual pelvic pain Adverse events (self-report)
(Cobellis et al*.*, 2011)	RCT	Italy	Age range 24–41; Endometriosis stage I and II	Micronized N-Palmitoylethanolamine (PEA) –transpolydatin supplement: 400 mg of PEA and 40 mg transpolydatin twice a day; *n* = 21	Placebo: One oral capsule twice a day; *n* = 20 Celecoxib (single course): 200 mg twice a day for 7 days; *n* = 20	All participants: conservative diagnostic surgery (pre-intervention)	3 months/post-intervention (EOI)	10 cm VAS Dysmenorrhoea, non-menstrual pelvic pain, dyspareunia
(Khodaverdi et al*.*, 2019)	RCT	Iran	Mean age 34 + 6; Baseline VAS score > 4; Endometriosis stage III-IV	Lactobacilus supplement: Lactobacillus acidophilus, Lactobacillus plantarum, Lactobacillus fermentum and Lactobacillus gasseri; One capsule daily; *n* = 19	Placebo: One capsule daily; *n* = 18	All participants: NSAIDs if required	8 weeks/post-intervention (EOI), 4 weeks post-intervention (ST)	10 cm VAS Dysmenorrhoea, non-menstrual pelvic pain, dyspareunia Adverse events (self-report)
(Kohama et al*.*, 2007)	Randomised comparative trial	Japan	Mean age 33 ± 4.0; Moderate—severe pelvic pain; Endometriosis stage II-IV	Pycnogenol supplement: French maritime pine bark extract; 30 mg twice a day via oral capsule; *n* = 32	Gn-RHa therapy: Leuprorelin acetate depot; 6 × 3.75 mg every 4 weeks; *n* = 26	All participants: conservative diagnostic surgery (< 6 months pre-intervention) Intervention: continue existing therapy, analgesics as required Comparator: Add-back therapy if required (Premarin, 1.25 mg per day)	48 weeks/post-intervention (EOI)	NRS (0–3) Dysmenorrhoea, non-menstrual pelvic pain Adverse events (self-report)
(Maia et al*.*, 2012)	Pre-post intervention study	Brazil	Mean age 30 ± 5; Experience pain and breakthrough bleeding	Resveratrol supplement (post-intervention): 30 mg once daily; *n* = 12	OC: 3 mg drospirenone + 30ug ethinylestradiol daily (pre-intervention); *n* = 12	Intervention: 3 mg drospirenone + 30ug ethinylestradiol	6 months/2 months post-intervention (ST)	NRS (0–3) Dysmenorrhoea
(Mares et al*.*, 2020)	RCT	France	Mean age: 34–36; Baseline VAS score (100 mm) > 40; Endometriosis stage II-IV	Metal trace elements supplement: Oral supplement taken daily; *n* = 32	Placebo: Oral capsule taken daily; *n* = 31	All participants: conservative diagnostic surgery (> 6 months pre-intervention); NSAIDs if required	120 days/post-intervention (EOI)	100 mm VAS Pain (overall) EHP-30 QoL NSAID use (self-report) Adverse events (self-report)
(Mendes da Silva et al*.*, 2017)	RCT	Brazil	Mean age 32–35	Resveratrol supplement: 40 mg a day; *n* = 22	Placebo: One oral capsule daily; *n* = 22	All participants OC: 0.15 mg levonorgestrel + 0.03 mg ethinyl estradiol	42 days/post-intervention (EOI)	10 cm VAS Pain (overall) Analgesic use (self-report) Adverse events (self-report)
(Morales-Prieto et al*.*, 2018)	Observational case series	Germany	Mean age 31–36; Baseline VAS score (100 mm) > 30	3,3'—Diindolylmethane (DIM) supplement: 100 mg three times a day for 3 months; *n* = 5	OC: 2 mg dienogest once a day for 3 months; *n* = 5	All participants: conservative diagnostic surgery (pre-intervention) Intervention: 2 mg dienogest once a day for 3 months	3 months/post-intervention (EOI)	100 mm VAS Non-menstrual pelvic pain Analgesic use (self-report)
(Nodler et al*.*, 2020)	RCT	USA	Mean age 19–20; Baseline VAS score (10 cm) > 3	Vitamin D supplement: 2000 IU Vitamin D3 daily via oral capsule; *n* = 27 Fish oil: 1000 mg fish oil daily via oral capsule; *n* = 20	Placebo: one oral capsule daily; *n* = 22	All participants: conservative diagnostic surgery (≥ 6 weeks pre-intervention); continue existing medical treatment	6 months/post-intervention (EOI)	10 cm VAS Pain (overall) SF-12 QoL: physical, mental Analgesic use (self-report)
(Schwertner et al*.*, 2013)	RCT	Brazil	Mean age 37–38; Baseline VAS score (10 cm) > 4; Endometriosis stage I-IV	Melatonin supplement: 10 mg once a day via oral capsule before bed; *n* = 20	Placebo: One capsule daily at bedtime; *n* = 20	All participants: supplementary analgesics (acetaminophen, ibuprofen, codeine, or tramadol) if required	8 weeks/post-intervention (EOI)	10 cm VAS Dysmenorrhoea, dyspareunia, pain (overall) Analgesic use (self-report)
*Dietary modifications*								
(Marziali et al*.*, 2012)	Retrospective observational case series	Italy	Median age 28 years; Baseline VAS score (10 cm) > 4	Gluten-free diet: complete removal of ingredients derived from gluten, including those within prescription medication, vitamins, cosmetics, stabilisers, and thickening agents. Patients were educated on the diet prior to partaking; *n* = 295	Baseline (pre-intervention); *n* = 295	All participants: conservative diagnostic surgery	12 months/post-intervention (EOI)	10 cm VAS (0 = no pain, 1–4 = mild, 5–7 = moderate, 8–10 = severe pain) Dysmenorrhoea, non-menstrual pelvic pain, dyspareunia
(Sesti et al*.*, 2007)	Randomised comparative trial	Italy	Mean age 29–31; Endometriosis stage III–IV	Dietary therapy dependent on BMI, physical activity, and job: vitamins (B6, A, C, E), minerals salts (calcium, magnesium, selenium, zinc, iron), VSL3 lactic ferments (Bifidobacterium breve, Bifidobacterium longum, Bifidobacterium infantis, Lactobacillus acidophilus, Lactobacillus casei, Lactobacillus bulgaricus, Streptococcus thermophilus), and omega-3 and omega-6 fatty acids (fish oil), secured nutritional intake between 1600—2000 cal; *n* = 37	1- Placebo; *n* = 115 2—Gn-RHa: either tryptorelin or leuproelin, 3.75 mg every 28 days; *n* = 42 3—Continous monophasic OC: 0.03 mg ethynilestradiol + 0.75 mg gestogen daily; *n* = 40	All participants: conservative laparoscopic procedure (7 days pre-intervention)	6 months/6 months post-intervention (IT)	10 mm VAS Dysmenorrhoea, non-menstrual pelvic pain, deep dyspareunia SF-36 QoL: Physical functioning, role limitation [physical], pain, general health perception, vitality, social functioning, role limitation [emotional], and mental health Adverse events (self-report)
(Signorile et al*.*, 2018)	Non-randomised controlled trial	Italy	Mean age 34–35; Endometriosis stage IV	Dietary therapy 1: Two capsules daily containing 1002 mg linoleic acid (omega 3), 432 mg alpha linolenic acid (omega 3), 172.8 mg linoleic acid (omega 6), 200 mg quercetin, 20 mg nicotinamide, 400mcg 5-methyltetrahydrofolate calcium salt, 20 mg titrated turmeric, 19.5 mg titrated parthenium; *n* = 30 Dietary therapy 2: Two capsules daily containing linseed oil and 5-methyltetrahydrofolate calcium salt; *n* = 30	Placebo: 2 doses per day (one every 12 h); *n* = 30	All participants Diet restrictions: increase fibre 20–30%, and to increase food containing Omega 3; reduce milk and derivatives by 30%; reduce meat, gluten, caffeine, alcohol, chocolate, saturated fat, butter, and margarine by 50%; complete exclusion of soy, aloe, and oats	3 months/post-intervention (EOI)	10 cm VAS Dysmenorrhoea, non-menstrual pelvic pain, dyspareunia
*OTC medication*								
(Kauppila and Ronnberg, 1985)	Cross-over trial	Finland	Mean age 32–35; Moderate-severe menstrual distress	Naproxen: 2 tablets (275 mg naproxen sodium per tablet) taken orally at first sign of menstrual distress, followed by 1 tablet every 4–6 h as required; *n* = 11	Placebo: 2 tablets taken orally at first sign of menstrual distress, followed by 1 tablet every 4–6 h as required; *n* = 9	All participants: supplementary analgesics following two consecutive doses of study drug if required	4 menstrual cycles (2 cycles for each comparator)/post-intervention (EOI)	NRS (0–3) Dysmenorrhoea Analgesic use (self-report) Adverse events (self-report)
*Exercise*								
(Goncalves et al*.*, 2017)	RCT	Brazil	Mean age 35 ± 7; Baseline VAS score (10 cm) > 4	Hatha yoga program: 2-h session twice a week; *n* = 28	No yoga: continue standard treatment (medication and/or one physiotherapy session a week); *n* = 12	All participants: continue existing medical treatment	8 weeks/post-intervention (EOI)	10 cm VAS Pain (overall) EHP-30 QoL: Pain, control and powerlessness, emotional wellbeing, social support, self-image, work, relationship with children, sexual intercourse, doctor relationship, treatment, infertility

BMI, body mass index; EHP-30, endometriosis health profile-30; EOI, end of intervention; Gn-RHa, gonadotropin-releasing hormone agonist; OC, oral contraceptive; NSAID, non-steroidal anti-inflammatory drug; NRS, numeric rating scale; QoL, quality of life; RCT, randomised controlled trial; SF-12, short form-12; ST, short term; USA, United States of America; VAS, visual analogue scale

### Type of Self-Management Strategy and Comparator

The efficacy of a range of self-management strategies were evaluated, including dietary supplements (*n* = 10) [29, 31–33, 35–38, 40, 43], dietary modifications (*n* = 3) [30, 39, 41], over the counter (OTC) naproxen sodium (herein referred to as naproxen) (*n* = 1) [42], and a hatha yoga program (*n* = 1) [34]. Comparator groups included placebos (*n* = 10) [30–33, 35–38, 41, 42], hormonal therapies (*n* = 4) [29, 30, 40, 43], a non-steroidal anti-inflammatory drug (NSAID) (*n* = 1) [31], ‘standard treatment’ (*n* = 1) [34], and baseline data (*n* = 1) [39].

### Outcome Measures and Follow-Up

Endometriosis symptoms were assessed by self-reported dysmenorrhoea, non-menstrual pelvic pain, dyspareunia, and overall pain. Some studies assessed quality of life, side effects, and additional medication use. Reporting additional medication in this review was a deviation from our original protocol, implemented because it became evident during data extraction that reporting use of other medications would provide a more comprehensive review of self-management. Effect sizes (SMD and 95% CI) could be calculated for eight studies (see Supplementary File 3 for all SMD and 95% CI calculations) [29, 30, 32–34, 36, 38, 43]. Authors of seven studies were contacted to gain additional information to calculate effect sizes, however the required data were not obtained and therefore excluded from these calculations. Pain intensity was frequently assessed using the 10 cm [31–36, 38, 39, 41] or 100 mm [30, 37, 40] Visual Analogue Scale (VAS). Three studies used a numeric pain rating scale (0 – 3) [29, 42, 43]. Quality of life was assessed using a range of self-report questionnaires, including the Endometriosis Health Profile (EHP) -30 [34, 37], the 36-Item Short Form Survey (SF-36) [30], and the 12-Item Short Form Survey (SF-12) [38]. For follow-up, 13 studies assessed outcome measures at end of intervention [29, 31–42]. Three studies assessed outcomes at additional follow-up time points, ranging from four weeks to six months post-intervention [30, 36, 43].

### Risk of Bias

#### Randomised Studies

Of the 11 randomised trials, seven were assessed as high risk [29, 30, 34, 36–38, 40], and four with some concerns[31–33, 35] (Table 2). Common reasons for risk of bias were randomisation methods (*n*= 4) and concealment of intervention allocation (*n* = 4). Trial protocols or registrations for nine studies were not reported or located [39, 41–43]; therefore, selection of the reported results was not evaluated.

**Table 2:**
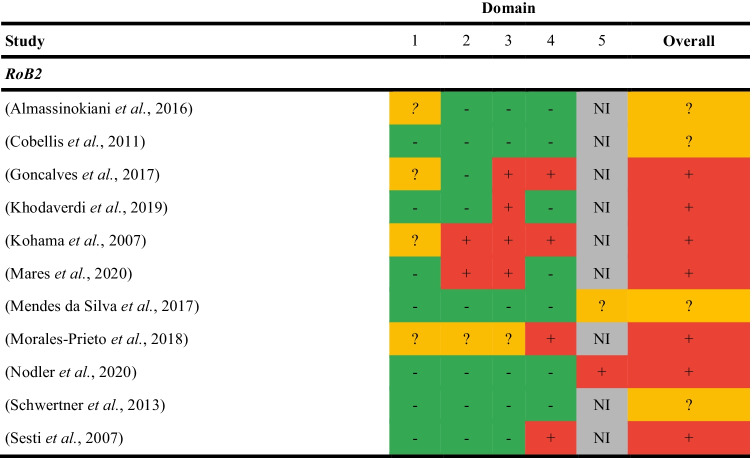
Risk of bias assessment for randomised studies

1- Randomisation; 2- Deviations from intended interventions; 3- Missing outcome data; 4- Measurement of outcome data; 5- Selection of the reported result

Green = low risk of bias; Yellow = some concerns; Red, high risk of bias

NI, no information; RoB2, Risk of Bias 2

#### Non-Randomised Studies

All non-randomised studies had a critical risk of bias overall (Table 3). Common reasons for being at risk of bias were confounding (*n* = 4) and blinding of participants (*n* = 3).

**Table 3:**
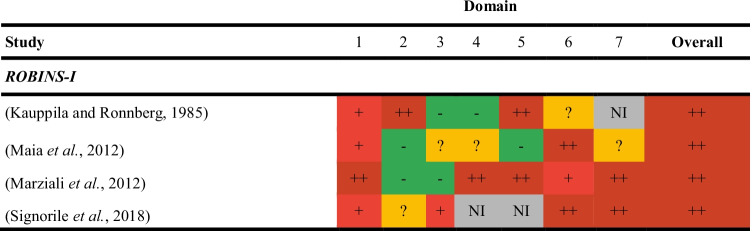
Risk of bias assessment for non-randomised studies

1- Confounding; 2- Selection of participants; 3- Classification of interventions; 4- Deviations from intended interventions; 5- Missing outcome data; 6- Measurement of outcome data; 7- Selection of the reported result

Green = low risk of bias; Yellow = moderate risk of bias; Light red = serious risk of bias; Dark red = critical risk of bias

NI, no information; ROBINS-I, Risk Of Bias In Non-randomized Studies – of Interventions

### Primary Outcomes

#### Dysmenorrhoea

**Fig. 2:**
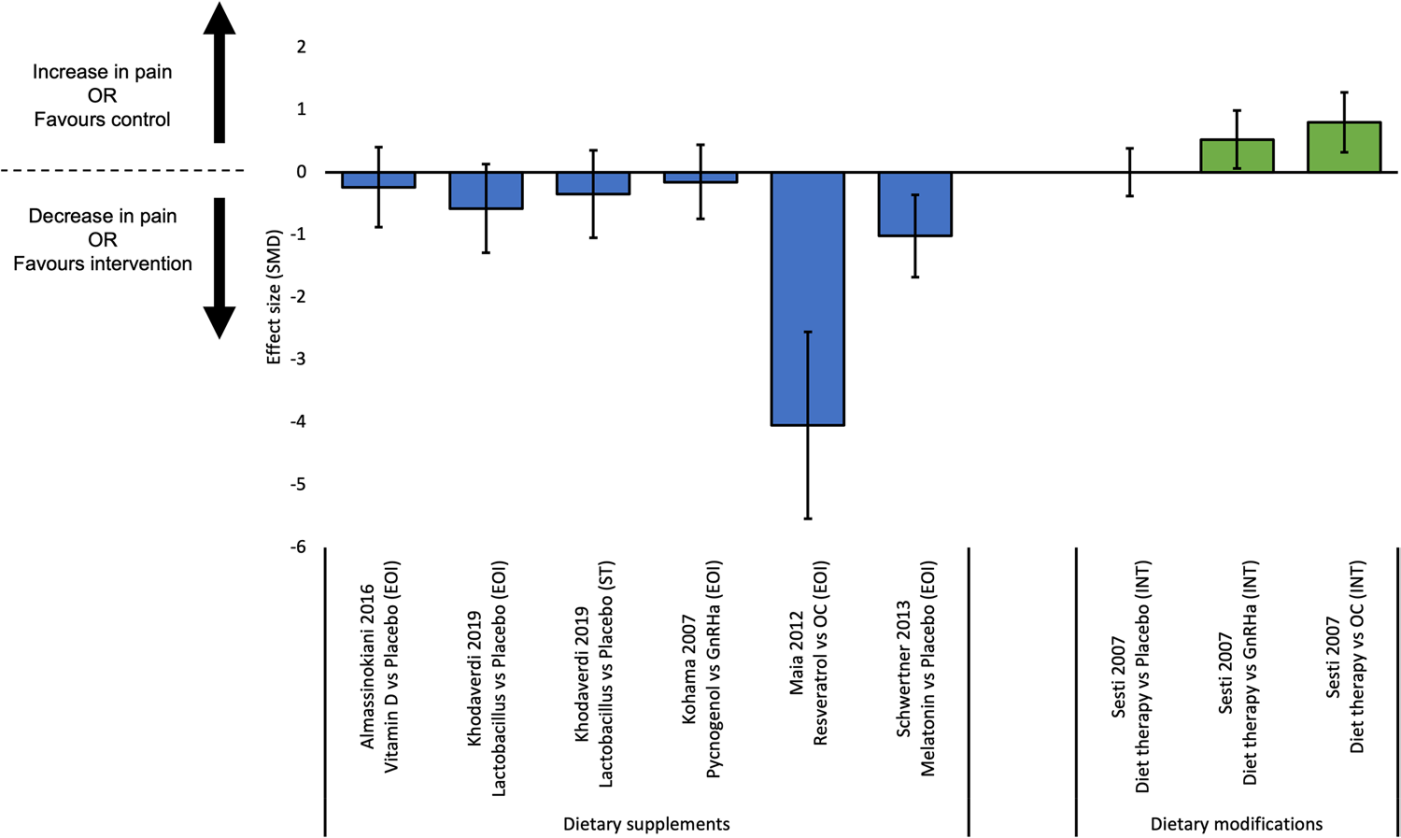
Effect sizes for studies evaluating continuous outcomes of dysmenorrhoea (standardised mean differences and 95% confidence intervals). Legend: EOI, end of intervention; GnRHa, gonadotropin releasing hormone agonist; IT, intermediate term; OC, oral contraceptive; SMD, standardised mean difference; ST, short term

Ten studies reported outcome data for dysmenorrhoea [29–33, 36, 39, 41–43]; effects sizes were calculated for nine comparisons from six studies (Fig. 2). Three RCTs compared dietary supplements (melatonin, Vitamin D, lactobacillus) to placebo [32, 33, 36]. Of those, melatonin was more effective than placebo at reducing dysmenorrhoea at end of intervention [32]. Conversely, Vitamin D was not more effective than placebo at reducing dysmenorrhoea at end of intervention [33], and lactobacillus was not more effective at end of intervention or 12-week post-intervention [36]. Two studies compared dietary supplements to hormonal therapies [29, 43]. In a pre-post intervention study of females with endometriosis using oral contraceptive, the addition of resveratrol in conjunction with the oral contraceptive was more effective at reducing dysmenorrhoea than oral contraceptive alone [43]. Additionally, one RCT found pycnogenol was not more effective than gonadotropin-releasing hormone agonist (Gn-RHa) [29]. For dietary modifications, one study found a diet protocol was not more effective than placebo, and was less effective than Gn-RHa and oral contraceptive at reducing dysmenorrhoea at six-month post-intervention [30].

Four studies reported outcome data for dysmenorrhoea that did not allow us to calculate effect sizes [31, 39, 41, 42]. One RCT found PEA-transpolydatin supplements (median 10 cm VAS score 3, range: 1.9 – 3.8) more effective than placebo (median 10 cm VAS score 5, range: 4.1 – 5.8), but less effective than a one-week course of celecoxib (median 10 cm VAS score 2.4, range: 1.4 – 3.2) at reducing dysmenorrhoea post-intervention [31]. For dietary modifications, a non-randomised controlled trial found the full dietary modification protocol had less percentage of participants with a ‘high’ intensity score (> 5 VAS score) for dysmenorrhoea following treatment (18%), compared to linseed oil and calcium salt supplements only (41%), or placebo (62%) [41]. Similarly, in a retrospective observational case series, 75% of participants reported a significant reduction in dysmenorrhoea following a gluten-free diet for 12 months (*p*-value < 0.005) [39]. In a cross-over trial, naproxen provided relief from dysmenorrhoea in a greater proportion of participants (83%) compared to placebo (41%) (*p*-value 0.008) [42].

#### Non-Menstrual Pelvic Pain

**Fig. 3:**
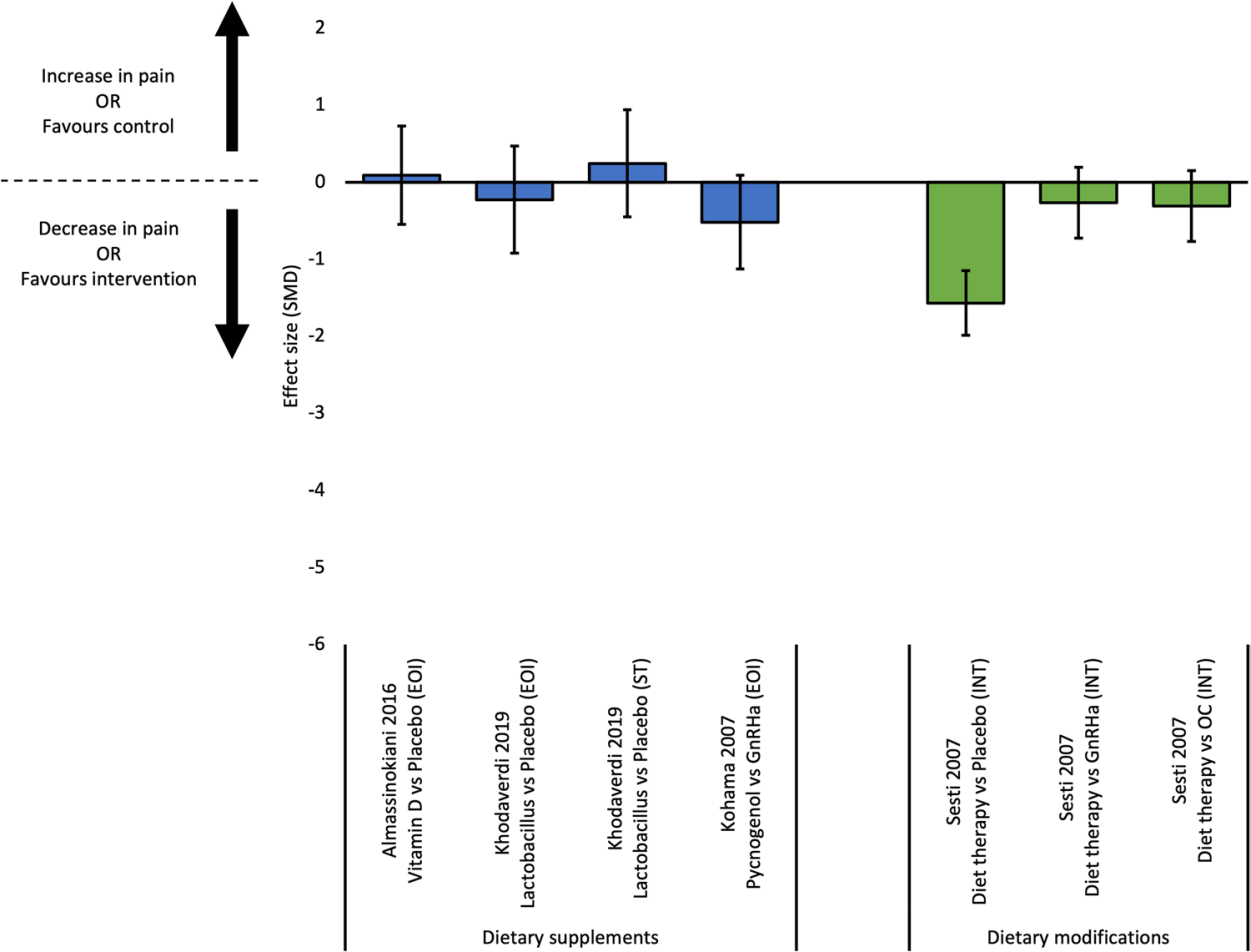
Effect sizes for studies evaluating continuous outcomes of non-menstrual pain (standardised mean differences and 95% confidence intervals). Legend: EOI, end of intervention; GnRHa, gonadotropin releasing hormone agonist; IT, intermediate term; OC, oral contraceptive; SMD, standardised mean difference; ST, short term

Eight studies reported outcome measures for non-menstrual pelvic pain [29–31, 33, 36, 39–41]. Effect sizes were calculated for seven comparisons from four studies (Fig. 3). Two RCTs compared dietary supplements to placebo; Vitamin D was not more effective than placebo at reducing non-menstrual pelvic pain at end of intervention [33], and lactobacillus was not more effective at end of intervention or 12-week post treatment [36]. Further, one RCT found pycnogenol was not more effective than Gn-RHa at reducing non-menstrual pelvic pain at end of intervention [29]. For dietary modifications, one RCT found a diet protocol more effective than placebo, but not more effective than Gn-RHa or oral contraceptive at reducing non-menstrual pelvic pain at six-month post-intervention [30].

Four studies reported outcome data for non-menstrual pelvic pain, where effect sizes could not be calculated [31, 39–41]. One RCT found PEA-transpolydatin supplements more effective than placebo (median 10 cm VAS score 4.8, range 3.9 – 5.5) but not more effective than a one-week course of celecoxib (median 10 cm VAS score 1.5, range 0.6 – 2.2) at reducing non-menstrual pelvic pain at end of intervention [31]. In an observational case series, DIM supplements combined with an oral contraceptive (median 100 mm VAS score 69.2, SD = 12.9) was not more effective than the oral contraceptive alone (median 100 mm VAS score 20.8, SD = 14.8) post-intervention [40]. For dietary modifications, a retrospective observational case series found 75% of participants significantly reduced painful symptoms following a gluten-free diet (*p*-value < 0.005) [39]. Similarly, a non-randomised controlled trial found dietary modifications had less percentage of participants with a ‘high’ intensity score (> 5 VAS score) for non-menstrual pelvic pain following treatment (18%), compared to linseed oil and calcium salt supplements only (45%), or placebo (60%) [41].

#### Dyspareunia

**Fig. 4:**
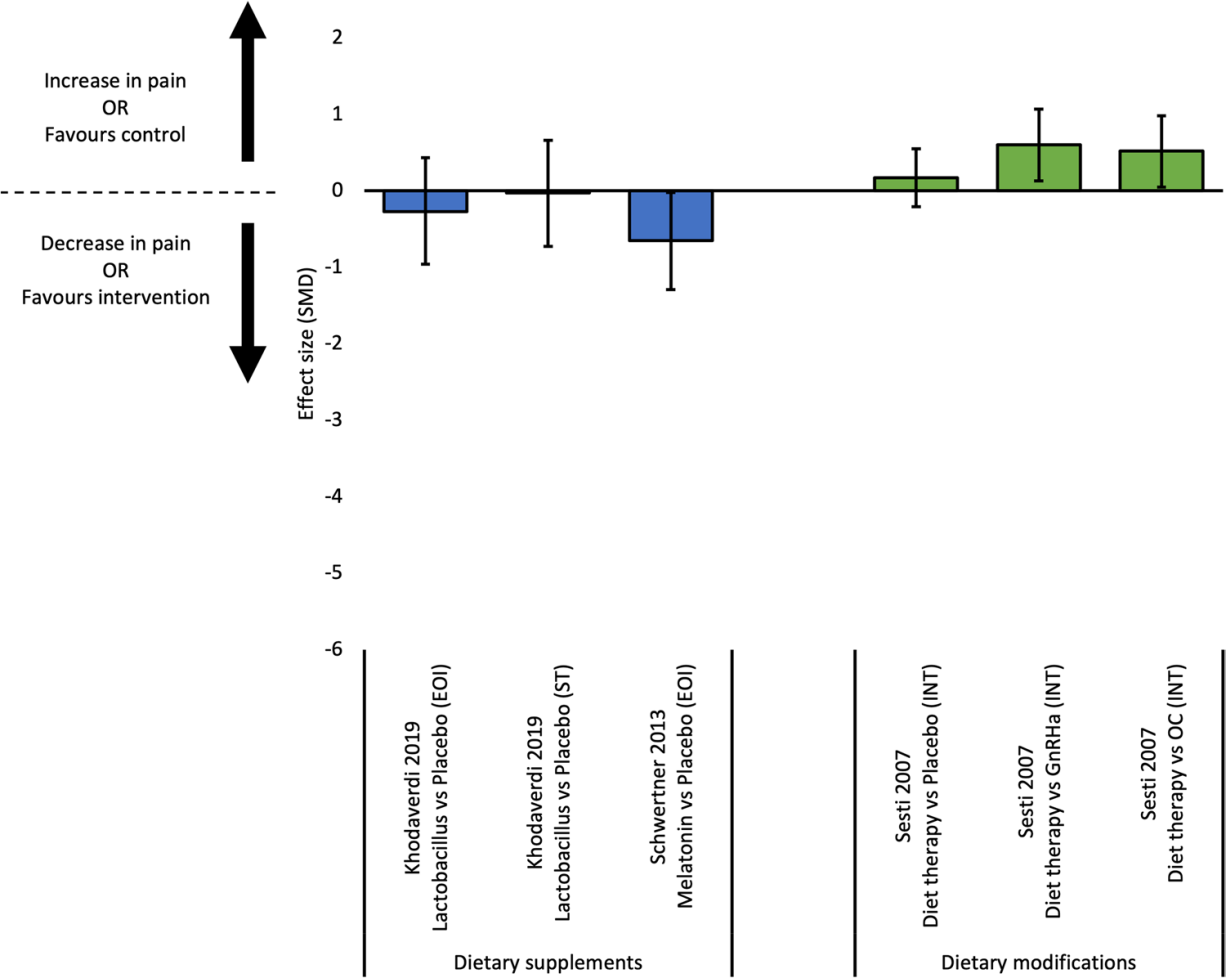
Effect sizes for studies evaluating continuous outcomes of dyspareunia (standardised mean differences and 95% confidence intervals). Legend: EOI, end of intervention; GnRHa, gonadotropin releasing hormone agonist; IT, intermediate term; OC, oral contraceptive; SMD, standardised mean difference; ST, short term

Six studies reported outcome data for dyspareunia [30–32, 36, 39, 41]. Effect sizes were calculated for six comparisons from three studies (Fig. 4). Two RCTs compared dietary supplements to placebo at end of intervention [32, 36]. Of those, melatonin was superior to placebo at reducing dyspareunia at end of intervention [32]. Conversely, lactobacillus supplements were not more effective than placebo at end of intervention or 12-weeks post-intervention[36]. A dietary modification protocol was not more effective than placebo, and less effective than Gn-RHa or oral contraceptive at reducing dyspareunia at six-month post-intervention [30].

Three studies reported outcome data for dyspareunia where effect sizes could not be calculated [31, 39, 41]. PEA-transpolydatin supplements (median 10 cm VAS score 2.4, range 1.5–3.4) were more effective than placebo (median 10 cm VAS score 3.8, range 32.7–4.8; *p*-value < 0.001) but less effective than celecoxib (median 10 cm VAS score 2.0 range 1.1–1.3; *p*-value < 0.001) at reducing dyspareunia at end of intervention [31]. A gluten-free diet was effective at reducing painful symptoms, including dyspareunia, in 75% of participants after 12 months (*p*-value < 0.005) [39]. A dietary protocol had less percentage of participants with a ‘high’ intensity score (> 5 VAS score) for dyspareunia following treatment (15%), compared to linseed oil and calcium salt supplements only (37%), or placebo (30%) [41].

#### Overall Pain

**Fig. 5:**
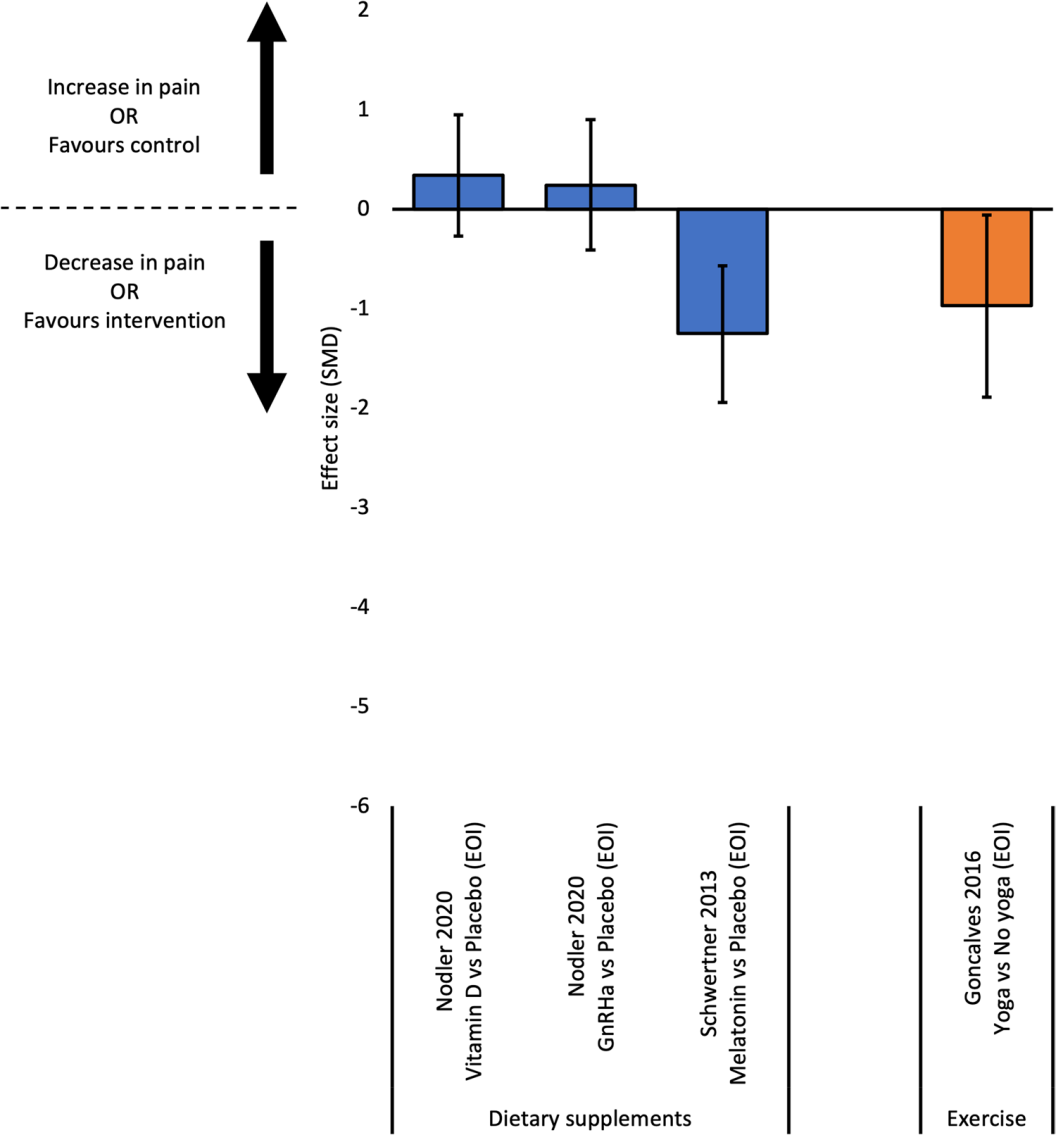
Effect sizes for studies evaluating continuous outcomes of overall pain (standardised mean differences and 95% confidence intervals). Legend: EOI, end of intervention; SMD, standardised mean difference

Five studies reported outcome data for overall pain [32, 34, 35, 37, 38], with effect sizes calculated for four comparisons from three studies (Fig. 5). Two RCTs compared dietary supplements to placebo at end of intervention [32, 38]. Melatonin was more effective than placebo at reducing overall pain [32]. Conversely, one RCT found both Vitamin D and fish oil supplements not more effective than placebo at reducing overall pain at end of intervention [38]. For exercise, one RCT found yoga more effective than no yoga at reducing overall pain at end of intervention [34].

Effect sizes could not be calculated for two RCTs investigating the efficacy of dietary supplements on overall pain [35, 37]. Metal trace element supplements (mean change from baseline 100 cm VAS -42.2, SD 5.1) were more effective than placebo (mean change from baseline 100 cm VAS -16.7, SD 5.4) at reducing overall pain intensity post-intervention (*p*-value < 0.001) [37]. Additionally, resveratrol supplements (median 10 cm VAS score 3.2, 95% CI 2.1 – 4.3) were not more effective than placebo (median 10 cm VAS score 3.9, 95% CI 2.2 – 5.0) at reducing overall pain post-intervention (*p*-value 0.7) [35].

### Secondary Outcomes

#### Quality of Life

Four studies evaluated quality of life (QoL) [34, 37, 38, 40]. Vitamin D, fish oil, and metal trace element supplements were not more effective than placebo at improving QoL at end of intervention [37, 38]. Dietary modifications were also not more effective than placebo, Gn-RHa, or oral contraceptive at six-month post-intervention [30]. Yoga significantly improved control and powerlessness, emotional wellbeing, self-image, work, and treatment QoL domains compared to no yoga at the end of the intervention (*p*-value < 0.05).

#### Use of Additional Medication

Seven studies reported the use of additional medication [29, 32, 35, 37, 38, 40, 42]. Melatonin supplements, metal trace element supplements, and naproxen were all associated with less reported use of analgesic medication compared to placebo [32, 37, 42]. Resveratrol, Vitamin D, and fish oil supplements were all associated with nonsignificant differences in the reported use of analgesic medication compared to placebo [35, 38]. No additional medication was reported for use with DIM and pycnogenol supplements [29, 40]. However, add-back therapy was used by eight participants within the Gn-RHa comparator group of the pycnogenol study [29].

#### Adverse Events

Seven studies collected outcome data for number of adverse events [29, 30, 33, 35–37, 42]. Resveratrol supplements were associated with similar adverse events reported for placebo [35]. Metal trace element supplements were associated with a similar number of adverse events compared to placebo [37]. Pycnogenol supplements were associated with various adverse events but differed from those reported in the Gn-RHa comparator arm [29]. Similarly, naproxen was associated with adverse events but differed from those for placebo [42]. One diet modification protocol was not associated with adverse events, however adverse events were associated with the Gn-RHa and oral contraceptive comparator arms [30]. Lactobacillus and melatonin supplements were not associated with adverse events [32, 36].

## Discussion

Most included studies demonstrate no significant effect of self-management strategies compared to placebo or other interventions for endometriosis symptoms. Where self-management strategies demonstrated efficacy, the findings remain unclear due to the sparcity and poor quality of evidence.

### Interpretation

Most dietary supplements were no more effective than placebo or frequently recommended medical interventions, at reducing pain-related outcomes. Paucity and poor quality of evidence identified in this review is in line with a previous review of supplements for endometriosis [44]. Importantly, dietary supplement regulation is less strict than standard pharmaceuticals [45], and impurities and suboptimal preparation of such supplements can contribute to adverse events [45]. Therefore, patient safety should also be considered when using supplements for managing endometriosis. No conclusive recommendations can be made regarding the use of dietary supplements for reducing endometriosis symptoms.

The efficacy of dietary *modifications* on reducing endometriosis symptoms is also unclear, with variable results. A previous review suggested dietary modifications (e.g. low-FODMAP and antioxidant diets) may help alleviate endometriosis symptoms [46]. However, most of the data included in that review were taken from within-group comparisons or qualitative studies, and evaluated outcomes unrelated to pain (e.g. vitamin intake), which is arguably the most bothersome symptom of endometriosis [47]. Given that females with endometriosis report various levels of improvement with dietary modifications [16], and the limited research in this area, it may be prudent to emphasise the common attributes shared between diet protocols, such as improved diet quality and increased nutrient density [48]. Recommendations for more specific diet-related interventions to reduce endometriosis-associated symptoms, seem premature.

Similar to other reviews [49], this review found that evidence for NSAIDs reducing endometriosis-associated symptoms is inconclusive. A single study suggests naproxen, an NSAID available OTC in many countries, may be an effective self-management strategy for reducing endometriosis-associated symptoms [42]. Naproxen acts by inhibiting the production of prostaglandins [50], which are often upregulated in the pathogenesis of endometriosis [51] and pain [52]. Considering the lack of evidence, it is surprising that naproxen, and other NSAIDs, are recommended in endometriosis clinical guidelines [53, 54]. High-quality empirical evidence is required to better understand the efficacy of naproxen for the management of endometriosis symptoms.

One study in this review assessed an active intervention – hatha yoga. That RCT found hatha yoga exercise to be more effective at reducing pain and improving quality of life in females with endometriosis than not performing yoga [34]. Uptake of active strategies by females with endometriosis, including exercise, is low, which may be due to the impact of symptoms [6] and lack of guidance from health professionals [24]. It may also be that the evidence supporting active interventions is limited and inconsistent. For example, although the wider pain literature suggests exercise is beneficial for persistent pain conditions [55], a systematic review of exercise for females with endometriosis suggests exercise has limited benefit [56] and may exacerbate pain symptoms [16]. The current state of evidence suggests it is premature to make claims regarding the efficacy of yoga for the management of endometriosis.

Much of the evidence evaluating self-management strategies for endometriosis is clouded by methodological limitations. Participant blinding of active strategies is difficult to overcome. Recent developments in blinding [57] and control treatments for studies of complex interventions [58] exist, and should be considered in future studies. It would also be prudent to evaluate potential mediators of outcome, potentially affected by non-blinding. Other risks of bias include missing outcome data and poor reporting of results, primarily due to the large number of dropouts and the likelihood of selecting specific outcomes from multiple analyses and/or participant subgroups. It should also be noted that the efficacy of self-management strategies for endometriosis may not be accurately reflected in trial settings. The use of self-management strategies in clinical practice is often aimed at reducing the most important symptom, and this becomes difficult in trials comprising a heterogeneous population of people with endometriosis with varied complaints. Pre-registered protocols were unavailable for most studies included in this review, despite their endorsement for transparency in reporting across pain research [59].

### Strengths and Limitations

There are several strengths to this review. We developed a comprehensive search strategy with search terms for passive and active self-management strategies, and commonly reported strategies. We prospectively registered the review. We reported the review in line with PRISMA and we ensured screening in duplicate, risk of bias evaluation and data extraction. Finally, we included a wide range of study designs.

This review also has limitations. High heterogeneity between studies meant we were unable to compare effect estimates across studies. The lack of a ‘gold standard’ definition for ‘self-management’ meant we had to devise our own set of criteria for a self-management strategy. We have therefore excluded interventions that might be considered ‘self-management’ under a different definition (e.g. acupuncture, massage). We only included studies with participants diagnosed with endometriosis via laparoscopy or histological confirmation because other common methods of diagnosis are unreliable. We also chose to include only those studies published in English, and therefore may have missed relevant records in other languages.

### Future Research Recommendations

Considering the methodological and evidence-base limitations highlighted in this review, we propose recommendations for future research. First, high-quality empirical evidence using a core outcome set will help to better understand the efficacy of self-management strategies for females with endometriosis and reduce study heterogeneity. Finally, a better understanding of facilitators of, and barriers to, self-management in females with endometriosis may improve the care and management of endometriosis in a clinical setting, and therefore patient outcomes.

## Conclusion

Many self-management strategies for endometriosis symptoms evaluated in this review demonstrate no significant effect on self-reported outcomes associated with endometriosis symptoms, when compared to placebo or hormonal therapies. Findings cannot be generalised due to limited evidence, study heterogeneity, and a high to critical risk of bias across the body of evidence. Further research investigating the efficacy of self-management strategies for females with endometriosis is required, so recommendations regarding their use can be made, and ultimately improve patient outcomes.

## Supplementary Information

Below is the link to the electronic supplementary material.

Supplementary file1 (PDF 104 KB)

Supplementary file2 (PDF 117 KB)

Supplementary file3 (PDF 73 KB)
