# Supplementary material for: The Efficacy of Self-Management Strategies for Females with Endometriosis: a Systematic Review
Source: Reprod Sci. 2022 Apr 29;30(2):390–407. doi: 10.1007/s43032-022-00952-9 (PMC9988721; doi:10.1007/s43032-022-00952-9)
Supplement: Supplementary file 1 — Supplementary file1 (PDF 104 KB) [file 43032_2022_952_MOESM1_ESM.pdf]

Supplementary file 1: Search strategy

Emcare (943 hits)

1	endometriosis/
2	endometrio*.tw.
3	1 or 2
4	self care/
5	self help/
6	self medication/
7	((self or selves) adj2 (care or manage* or medicat*)).tw.
8	or/4-7
9	exp diet/
10	(gluten adj3 diet).tw.
11	(fodmap adj3 diet).tw.
12	dietary supplement/
13	(diet adj2 supplement).tw.
14	antioxidant/
15	antioxidant*.tw.
16	vitamin/
17	vitamin*.tw.
18	chinese medicine/
19	medicinal plant/
20	plant extract/
21	(Chinese adj2 (herb* or plant* or medicin*)).tw.
22	(plant* adj3 (medicin* or extract*)).tw.
23	or/9-22
24	exp exercise/
25	exp kinesiotherapy/
26	exp yoga/
27	exercise$.tw.
28	aerobic$.tw.
29	(yoga or walk* or gym or crossfit or circuit or swim* or jog* or run* or train* or 'tai chi').tw.
30	((muscle adj3 stretch*) or stretch* or mobility).tw.
31	((weight adj3 (lifting or train*)) or (resistan* adj3 train*)).tw.
32	(physical adj3 activ*).mp.
33	or/24-32
34	exp meditation/
35	relaxation training/
36	exp mindfulness/
37	breathing exercise/
38	aromatherapy/
39	(meditat* or relax* or mindful* or breath* or aromatherap*).tw.
40	((meditat* or relax* or mindful* or breath*) adj3 (therap* or exercis*)).tw.

Supplementary file 1: Search strategy

41 (stress adj3 (manag* or reduc*)).tw.
42 or/34-41
43 non prescription drug/
44 nonsteroid antiinflammatory agent/
45 paracetamol/
46 acetylsalicylic acid/
47 ibuprofen/
48 naproxen/
49 mefenamic acid/
50 ((nonprescription adj2 drug$) or ('over the counter' adj2 drug$)).tw.
51 (nonsteroidal$ or non-steroidal$ or nsaid$).tw.
52 (aspirin or ibuprofen or naprogesic or mefenamic acid or ponstan or voltaren or naproxen or paracetamol or acetaminophen).tw.
53 or/43-52
54 transcutaneous electrical nerve stimulation/
55 (('transcutaneous electric nerve' adj2 stimulat*) or tens).mp. or ('electric* nerve' adj2 stimulat*).tw.
56 54 or 55
57 high temperature/
58 cold/
59 ((hot adj2 temperature) or heat or (wheat adj2 (pack or bag)) or ('hot water' adj2 bottle)).tw.
60 ((cold adj2 temperature) or cold or (ice adj2 pack)).tw.
61 57 or 58 or 59 or 60
62 8 or 23 or 33 or 42 or 53 or 56 or 61
63 3 and 62

Mardon AK, Leake HB, Hayles C, Henry M, Neumann T, Moseley GL, Chalmers KJ
Corresponding author: k.jane.chalmers@gmail.com

Supplementary file 1: Search strategy

Embase (3955 hits)

1	endometriosis/
2	endometrio*.mp.
3	1 or 2
4	self care/
5	self medication/
6	self-management/
7	((self or selves) adj2 (care or manage* or medicat*)).tw.
8	or/4-7
9	exp diet/
10	(gluten adj3 diet).tw.
11	(fodmap adj3 diet).tw
12	dietary supplement/
13	(diet adj2 supplement*).tw.
14	Antioxidants/
15	antioxidant*.tw.
16	Vitamins/
17	vitamin*.tw.
18	Chinese medicine/
19	medicinal plant/
20	plant extract/
21	(Chinese adj2 (herb* or plant* or medicin*)).tw.
22	(plant* adj3 (medicin* or extract*)).tw.
23	or/9-22
24	exp exercise/
25	exp kinesiotherapy/
26	exp yoga/
27	exercise$.tw.
28	aerobic$.tw.
29	(yoga or walk* or gym or crossfit or circuit or swim* or jog* or run* or train* or 'tai chi').tw.
30	((muscle adj3 stretch*) or stretch* or mobility).tw.
31	((weight adj3 (lifting or train*)) or (resistan* adj3 train*)).tw.
32	(physical adj3 activ*).tw.
33	or/24-32
34	exp meditation/
35	relaxation training/
36	exp mindfulness/
37	breathing exercise/
38	aromatherapy/
39	(meditat* or relax* or mindful* or breath* or aromatherap*).tw.
40	((meditat* or relax* or mindful* or breath*) adj3 (therap* or exercis*)).tw.

Mardon AK, Leake HB, Hayles C, Henry M, Neumann T, Moseley GL, Chalmers KJ
Corresponding author: k.jane.chalmers@gmail.com

Supplementary file 1: Search strategy

41 (stress adj3 (manag* or reduc*)).tw.
42 or/34-41
43 non prescription drug/
44 nonsteroid antiinflammatory agent/
45 paracetamol/
46 acetylsalicylic acid/
47 ibuprofen/
48 naproxen/
49 mefenamic acid/
50 ((nonprescription adj2 drug$) or ('over the counter' adj2 drug$)).tw.
51 (nonsteroidal$ or non-steroidal$ or nsaid$).tw.
52 (aspirin or ibuprofen or naprogesic or ponstan or ‘mefenamic acid ‘or voltaren or naproxen or paracetamol or acetaminophen).tw.
53 or/43-52
54 transcutaneous electrical nerve stimulation/
55 (('transcutaneous electric nerve' adj2 stimulat*) or tens).mp. or ('electric* nerve' adj2 stimulat*).tw.
56 54 or 55
57 high temperature/
58 cold/
59 ((hot adj2 temperature) or heat or (wheat adj2 (pack or bag)) or ('hot water' adj2 bottle)).tw
60 ((cold adj2 temperature) or cold or (ice adj2 pack)).tw.
61 57 or 58 or 59 or 60
62 8 or 23 or 33 or 42 or 53 or 61
63 3 and 6

Mardon AK, Leake HB, Hayles C, Henry M, Neumann T, Moseley GL, Chalmers KJ
Corresponding author: k.jane.chalmers@gmail.com

Supplementary file 1: Search strategy

MEDLINE (1683 hits)

1	Endometriosis/
2	endometrio*.mp.
3	1 or 2
4	Self-Management/
5	Self Care/
6	Self Medication/
7	((self or selves) adj2 (care or manage* or medicat*)).tw,kf.
8	or/4-7
9	exp Diet/
10	Diet, Gluten-Free/
11	(gluten adj3 diet).tw,kf.
12	(fodmap adj3 diet).tw,kf.
13	exp Dietary Supplements/
14	(diet* adj2 supplement*).tw,kf.
15	Antioxidants/
16	antioxidant*.tw,kf.
17	Vitamins/
18	vitamin*.tw,kf.
19	Drugs, Chinese Herbal/
20	Plants, Medicinal/
21	Plant Extracts/
22	(Chinese adj2 (herb* or plant* or medicin*)).tw,kf.
23	(plant* adj3 (medicin* or extract*)).tw,kf.
24	or/9-23
25	exp Exercise/
26	exp Exercise Therapy/
27	Yoga/
28	exercise$.tw,kf.
29	aerobic$.tw,kf.
30	(yoga or walk* or gym or crossfit or circuit or swim* or jog* or run* or train* or 'tai chi').tw,kf.
31	((muscle adj3 stretch*) or stretch* or mobility).tw,kf.
32	((weight adj3 (lifting or train*)) or (resistan* adj3 train*)).tw,kf.
33	(physical adj3 activ*).tw,kf.
34	or/25-33
35	Meditation/
36	Relaxation Therapy/
37	Mindfulness/
38	Breathing Exercises/
39	Aromatherapy/
40	(meditat* or relax* or mindful* or breath* or aromatherap*).tw,kf.

Supplementary file 1: Search strategy

41	((meditat* or relax* or mindful* or breath*) adj3 (therap* or exercis*)).tw,kf.
42	(stress adj3 (manag* or reduc*)).tw,kf.
43	or/35-42
44	Nonprescription Drugs/
45	Anti-Inflammatory Agents, Non-Steroidal/
46	Acetaminophen/
47	Aspirin/
48	Ibuprofen/
49	Naproxen/
50	mefenamic acid/
51	((nonprescription adj2 drug$) or ('over the counter' adj2 drug$)).tw,kf.
52	(nonsteroidal$ or non-steroidal$ or nsaid$).tw,kf.
53	(aspirin or ibuprofen or naprogesic or ‘mefenamic acid’ or ponstan or voltaren or naproxen or paracetamol or acetaminophen).tw,kf.
54	or/44-53
55	Transcutaneous Electric Nerve Stimulation/
56	((('transcutaneous electric nerve' adj2 stimulat*) or tens) or ('electric* nerve' adj2 stimulat*)).tw,kf.
57	55 or 56
58	Hot Temperature/
59	Cold Temperature/
60	((hot adj2 temperature) or heat or (wheat adj2 (pack or bag)) or ('hot water' adj2 bottle)).tw,kf.
61	((cold adj2 temperature) or cold or (ice adj2 pack)).tw,kf.
62	58 or 59 or 60 or 61
63	8 or 24 or 34 or 43 or 54 or 57 or 62
64	3 and 63

Mardon AK, Leake HB, Hayles C, Henry M, Neumann T, Moseley GL, Chalmers KJ
Corresponding author: k.jane.chalmers@gmail.com

The efficacy of self-management strategies for females with endometriosis: a systematic review
Reproductive Sciences

Supplementary file 1: Search strategy

Scopus (2989 hits)

(TITLE-ABS-KEY (endometrio*) AND TITLE-ABS-KEY ("self care" OR "self help" OR self-management) OR TITLE-ABS-KEY ((self OR selves) W/1 (care OR manage* OR medicat*)) OR TITLE-ABS-KEY (diet OR (gluten W/3 diet) OR (fodmap W/3 diet) OR (dietary W/1 supplement*) OR (diet* W/2 supplement*) OR antioxidant* OR vitamin* OR (chinese W/1 medicine) OR (medicin* W/1 plant) OR (plant W/1 extract) OR (chinese W/2 (herb* OR plant* OR medicin*)) OR (plant* W/3 (medicin* OR extract*))) OR TITLE-ABS-KEY (exercise* OR (exercise W/2 therapy) OR kinesiotherap* OR yoga OR aerobic* OR walk* OR gym OR crossfit OR circuit OR swim* OR jog* OR run* OR train* OR (tai W/1 chi) OR ((muscle W/3 stretch*) OR stretch* OR mobility) OR ((weight W/3 (lifting OR train*)) OR (resistan* W/3 train*)) OR (physical W/3 activ*)) OR TITLE-ABS-KEY (meditat* OR (relax* W/2 therapy) OR mindful* OR (breath* W/2 exercise*) OR aromatherapy OR (stress W/3 (manag* OR reduc*))) OR TITLE-ABS-KEY (“non AND prescription” OR “non-prescription” W/2 drug OR ("over the counter" W/2 drug)) OR TITLE-ABS-KEY (“nonsteroidal” OR “non-steroidal” W/1 antiinflammator* OR “anti-inflammatory” OR “anti-inflammatories”) OR TITLE-ABS-KEY (paracetamol OR aspirin OR ibuprofen OR naprogesic OR "mefenamic acid" OR ponstan OR voltaren OR naproxen OR acetaminophen) OR TITLE-ABS-KEY (“transcutaneous AND electrical AND nerve” W/1 stimulat* OR tens OR "electric* nerve" W/2 stimulat*) OR TITLE-ABS-KEY (hot W/2 temperature OR heat OR wheat W/2 (pack OR bag) OR "hot water" W/2 bottle OR cold W/2 temperature OR cold OR ice W/2 pack)

Web of Science (1497 results)

TOPIC: (endometrio*)
AND TOPIC: (((self OR selves) NEAR/1 (care OR manage* OR medicat*)) OR diet OR ((gluten OR fodmap) NEAR/1 diet) OR diet NEAR/1 supplement OR antioxident* OR vitamin* OR (chinese NEAR/1 (herb* OR plant* OR medicin*)) OR (plant* NEAR/2 (medicin* OR extract*)) OR exercis$ OR aerobic$ OR yoga OR walk* OR gym OR crossfit OR circuit OR swim* OR jog* OR run* OR train* OR "tai chi" OR stretch* or mobility OR (weight NEAR/2 (lifting OR train*)) OR resistan* NEAR/2 train* OR physical NEAR/2 activ* OR meditat* OR relax* OR mindful* OR breath* OR aromatherap* OR (stress NEAR/2 (manag* OR reduc*)) OR nonprescription NEAR/1 drug$ OR "non prescription" NEAR/1 drug$ OR "over the counter" NEAR/1 drug$ OR nonsteroidal$ OR "non steroidal$" OR nsaid$ OR aspirin OR ibuprofen OR naprogesic OR "mefenamic acid" OR ponstan OR voltaren OR naproxen OR paracetamol OR acetaminophen OR "transcutaneous electric nerve" NEAR/1 stimulat* OR tens OR "electric* nerve" NEAR/1 stimulat* OR hot NEAR/1 temperature OR heat OR (wheat NEAR/1 (pack OR bag)) OR "hot water" NEAR/1 bottle OR cold NEAR/1 temperature OR ice NEAR/1 pack)

Mardon AK, Leake HB, Hayles C, Henry M, Neumann T, Moseley GL, Chalmers KJ
Corresponding author: k.jane.chalmers@gmail.com

The efficacy of self-management strategies for females with endometriosis: a systematic review
Reproductive Sciences

Supplementary file 1: Search strategy

CENTRAL (344 hits)

#1	MeSH descriptor: [Endometriosis] explode all trees
#2	endometrio*
#3	#1 OR #2
#4	MeSH descriptor: [Self-Management] this term only
#5	MeSH descriptor: [Self Care] this term only
#6	MeSH descriptor: [Self Medication] this term only
#7	(self or selves) near/1 (care or manage* or medicat*)
#8	#4 OR #5 OT #6 OR #7
#9	MeSH descriptor: [Diet] explode all trees
#10	MeSH descriptor: [Diet, Gluten-Free] this term only
#11	((gluten OR fodmap) NEAR/1 diet)
#12	MeSH descriptor: [Dietary Supplements] explode all trees
#13	(diet* NEAR/2 supplement*)
#14	MeSH descriptor: [Antioxidants] this term only
#15	antioxidant*
#16	MeSH descriptor: [Vitamins] this term only
#17	vitamin*
#18	MeSH descriptor: [Drugs, Chinese Herbal] this term only
#19	MeSH descriptor: [Plants, Medicinal] this term only
#20	MeSH descriptor: [Plant Extracts] this term only
#21	(chinese NEAR/1 (herb* OR plant* OR medicin*)) OR (plant* NEAR/2 (medicin* OR extract*))
#22	#9 OR #10 OR #11 OR #12 OR #13 OR #14 OR #15 OR #16 OR #17 OR #18 OR #19 OR #20 OR #21
#23	MeSH descriptor: [Exercise] explode all trees
#24	MeSH descriptor: [Exercise Therapy] explode all trees
#25	MeSH descriptor: [Yoga] this term only
#26	exercis$ OR aerobic$ OR yoga OR walk* OR gym OR crossfit OR circuit OR swim* OR jog* OR run* OR train* OR "tai chi" OR stretch* or mobility OR (weight NEAR/2 (lifting OR train*)) OR resistan* NEAR/2 train* OR physical NEAR/2 activ*
#27	#23 OR #24 OR #25 OR #26
#28	MeSH descriptor: [Meditation] this term only
#29	MeSH descriptor: [Relaxation Therapy] this term only
#30	MeSH descriptor: [Mindfulness] this term only
#31	MeSH descriptor: [Breathing Exercises] this term only
#32	meditat* OR relax* OR mindful* OR breath* OR aromatherap* OR (stress NEAR/2 (manag* OR reduc*))
#33	#28 OR #29 OR #30 OR #31 #32
#34	MeSH descriptor: [Nonprescription Drugs] this term only
#35	MeSH descriptor: [Anti-Inflammatory Agents, Non-Steroidal] explode all trees
#36	MeSH descriptor: [Acetaminophen] this term only
#37	MeSH descriptor: [Aspirin] this term only

Mardon AK, Leake HB, Hayles C, Henry M, Neumann T, Moseley GL, Chalmers KJ
Corresponding author: k.jane.chalmers@gmail.com

Supplementary file 1: Search strategy

#38 MeSH descriptor: [Ibuprofen] this term only
#39 MeSH descriptor: [Naproxen] this term only
#40 MeSH descriptor: [Mefenamic Acid] this term only
#41 nonprescription NEAR/1 drug$ OR "non prescription" NEAR/1 drug$ OR "over the counter" NEAR/1 drug$ OR nonsteroidal$ OR "non steroidal$" OR nsaid$ OR aspirin OR ibuprofen OR naprogesic OR "mefenamic acid" OR ponstan OR voltaren OR naproxen OR paracetamol OR acetaminophen
#42 #34 OR #35 OR #36 OR #37 OR #38 OR #39 OR #40 OR #41
#43 MeSH descriptor: [Transcutaneous Electric Nerve Stimulation] this term only
#44 "transcutaneous electric nerve" NEAR/1 stimulat* OR tens OR "electric* nerve" NEAR/1 stimulat*
#45 #43 OR #44
#46 MeSH descriptor: [Hot Temperature] this term only
#47 MeSH descriptor: [Cold Temperature] this term only
#48 hot NEAR/1 temperature OR heat OR (wheat NEAR/1 (pack OR bag)) OR "hot water" NEAR/1 bottle OR cold NEAR/1 temperature OR ice NEAR/1 pack
#49 #46 OR #47 OR #48
#50 #8 OR #22 OR #27 OR #33 OR #42 OR #45 OR #49
#51 #3 AND #50 (in trials)

Supplementary file 1: Search strategy

Google Scholar search terms

Endometriosis	AND self-management
	AND diet
	AND supplement
	AND Chinese medicine
	AND plant extract
	AND exercise
	AND yoga
	AND walking
	AND gym
	AND swimming
	AND running
	AND tai chi
	AND stretching
	AND meditation
	AND mindfulness
	AND relaxation
	AND non-prescription drugs
	AND nonsteroidal anti-inflammatories
	AND paracetamol
	AND aspirin
	AND ibuprofen
	AND naprogesic
	AND mefenamic acid
	AND transcutaneous electrical nerve stimulator
	AND heat
	AND hot pack
	AND cold

The efficacy of self-management strategies for females with endometriosis: a systematic review
Reproductive Sciences

Supplementary file 1: Search strategy

Websites of organisations
Endometriosis Australia https://www.endometriosisaustralia.org/
Endometriosis.org https://endometriosis.org/
Endometriosis Foundation of America https://www.endofound.org/
Endometriosis Association https://endometriosisassn.org/
Speakendo.com https://www.speakendo.com/
QENDO https://www.qendo.org.au/
EndoActive https://endoactive.org.au/
Endometriosis UK https://www.endometriosis-uk.org/
Australian Coalition for Endometriosis https://www.acendo.com.au/
Endometriosis New Zealand https://nzendo.org.nz/
World Endometriosis Society https://endometriosis.ca/b
The Society of Endometriosis and Uterine Disorders https://seud.org/
World Endometriosis Research Foundation https://endometriosisfoundation.org/
Endometriosis Association of Ireland https://www.endometriosis.ie/
Endometriosis Research Centre https://www.endocenter.org/

Reviews sought for literature via reference lists
Psychological and mind-body interventions for endometriosis: A systematic review (Evans *et al.*, 2019)
Endometriosis and physical exercises: a systematic review (Bonocher *et al.*, 2014)
Systematic review and meta-analysis of complementary treatments for women with symptomatic endometriosis (Mira *et al.*, 2018)
Self-management in condition-specific health: a systematic review of the evidence among women diagnosed with endometriosis (O'Hara *et al.*, 2019)
Non-surgical interventions for the management of chronic pelvic pain (Cheong *et al.*, 2014)
Effectiveness of Dietary Interventions in the Treatment of Endometriosis: a Systematic Review (Nirgianakis *et al.*, 2021)
The effects of nutrients on symptoms in women with endometriosis: a systematic review (Huijs and Nap, 2020)

Guidelines sought for literature via reference lists
Australian clinical practice guideline for the diagnosis and management of endometriosis (RANZCOG, 2021)
Clinical evaluation and management of endometriosis: guideline for Korean patients from Korean Society of Endometriosis (Hwang et al., 2018)
Endometriosis: diagnosis and management (NICE, 2017)
ESHRE guideline: management of women with endometriosis (Dunselman et al., 2014)
Management of endometriosis CNGOF/HAS clinical practice guidelines - Short version (Collinet et al., 2018)
National German Guideline (S2k): Guideline for the Diagnosis and Treatment of Endometriosis (Ulrich et al., 2014)
ACOG Management of endometriosis (ACOG, 2021)

Mardon AK, Leake HB, Hayles C, Henry M, Neumann T, Moseley GL, Chalmers KJ
Corresponding author: k.jane.chalmers@gmail.com

The efficacy of self-management strategies for females with endometriosis: a systematic review
Reproductive Sciences

Supplementary file 1: Search strategy

Mardon AK, Leake HB, Hayles C, Henry M, Neumann T, Moseley GL, Chalmers KJ
Corresponding author: k.jane.chalmers@gmail.com
