# Supplementary material for: The Efficacy of Self-Management Strategies for Females with Endometriosis: a Systematic Review
Source: Reprod Sci. 2022 Apr 29;30(2):390–407. doi: 10.1007/s43032-022-00952-9 (PMC9988721; doi:10.1007/s43032-022-00952-9)
Supplement: Supplementary file 2 — Supplementary file2 (PDF 117 KB) [file 43032_2022_952_MOESM2_ESM.pdf]

The efficacy of self-management strategies for females with endometriosis: a systematic review
Reproductive Sciences

Supplementary file 2: Excluded full-text articles

Citation	Title	Reason for exclusion
Borghini 2018	Relationship between nickel allergic contact mucositis and nickel-rich diet in symptomatic women suffering from endometriosis	Wrong publication type
	Using a Herbal Remedy Extract for the Treatment of Endometriosis Symptoms	Wrong publication type
	The effect of garlic tablet on the endometriosis related pain in women	Wrong publication type
	Flexofytol® for the Treatment of Endometriosis- Associated Pain	Wrong publication type
	Iyengar Yoga Therapy for Dysmenorrhea and Endometriosis	Wrong publication type
Nodler 2018	Supplementation in adolescent girls with endometriosis (SAGE): a double blind, randomized, placebo-controlled trial	Wrong publication type
	Effect of Aspirin On treatment of endometriosis	Wrong publication type
Nodler 2018	Supplementation in adolescent girls with endometriosis (SAGE): a double blind, randomized, placebo controlled trial	Wrong publication type
Farshi 2020	Effect of self-care counselling on depression and anxiety in women with endometriosis: a randomized controlled trial	Wrong intervention
DeLeo 2019	Role of a natural integrator based on lipoic acid, palmitoiletanolamide and myrrh in the treatment of chronic pelvic pain and endometriosis	Wrong patient population
Marziali 2015	Role of Gluten-Free Diet in the Management of Chronic Pelvic Pain of Deep Infiltranting Endometriosis	Wrong publication type
Haines 2005	Treatment of endometriosis with acupuncture and other complementary therapies	Wrong publication type
Gomez 2013	Acupuncture and other complementary therapies in endometriosis	Wrong publication type
Tian 2004	Observation on curative effect on treating endometriosis with Xiao Yi Fang	Could not access
	Chinese herbs in the treatment of laparoscopy diagnosed endometriosis: a controlled study	Wrong publication type
Hansen 2017	Long-term effects of mindfulness-based psychological intervention for coping with pain in endometriosis: A six-year follow-up on a pilot study	Wrong patient population
	Effect of Mediterranean Diet and Physical Activity in Patients With Endometriosis	Wrong publication type
Nodler 2018	Supplementation in adolescent girls with endometriosis (SAGE): A double blind, randomized, placebo-controlled trial	Wrong publication type
	The effect of sensate focus technique and position changing on sexual function in women with pelvic endometriosis	Wrong publication type
	Electrotherapy in the treatment of pain during intercourse and assessment of the outcomes on the quality of life and sexuality of women with deep endometriosis	Wrong publication type

Mardon AK, Leake HB, Hayles C, Henry M, Neumann T, Moseley GL, Chalmers KJ
Corresponding author: k.jane.chalmers@gmail.com

The efficacy of self-management strategies for females with endometriosis: a systematic review
Reproductive Sciences

Supplementary file 2: Excluded full-text articles

Sesti 2009	Recurrence rate of endometrioma after laparoscopic cystectomy: a comparative randomized trial between post-operative hormonal suppression treatment or dietary therapy vs. placebo	Wrong outcome measures
Ferrero 2011	Letrozole and norethisterone acetate versus letrozole and triptorelin in the treatment of endometriosis related pain symptoms: A randomized controlled trial	Wrong intervention
Mira 2015	Effectiveness of complementary pain treatment for women with deep endometriosis through Transcutaneous Electrical Nerve Stimulation (TENS): randomized controlled trial	Other
Wilson 2020	Understanding the role of Facebook to support women with endometriosis: A Malaysian perspective	Wrong study design
Zhao 2013	Controlling the recurrence of pelvic endometriosis after a conservative operation: comparison between Chinese herbal medicine and western medicine	Wrong intervention
Han 2021	Medical intelligent processor system and traditional Chinese medicine to treat endometriosis	Wrong publication type
Bi 2018	Effect of neuromuscular electrical stimulation for endometriosis-Associated pain	Wrong intervention
Music 2005	Endometriosis and self-management strategies	Wrong publication type
	Comparing between prescription and not prescription of vitamin D on recurrence of endometriosis related pains	Wrong publication type
	The impact of vitamins and antioxidants on the severity of endometriosis, pelvic pain in women	Wrong publication type
	TENS effect and exercises in menstrual pain	Wrong publication type
	Efficacy of Trace Elements in the Treatment of Endometriosis: a Pilot Study	Wrong publication type
	TENS Self-applied in the Complementary Treatment of Deep Endometriosisthe e	Wrong publication type
Tuncer 2019	Effects on health and diet quality of the gluten free diet	Wrong publication type
	Eeffect of oral administration of N- acetyl cysteine in Women with Endometriosis	Wrong publication type
	Dietary Treatment of Endometriosis-related Irritable Bowel Syndrome	Wrong publication type
Cao 2008	Treatment of Pelvic Pain Caused by Endometriosis with Xuejie Huayu Zhitong Fang	Could not access
	Yoga in the management of pain and related stress on women with Endometriosis	Wrong publication type
	Evaluating the influence of Yoga, Cognitive Behaviour Therapy and Standard Care on Quality of Life and Healthcare Costs in Endometriosis	Wrong publication type
	DLBS1442 for The Treatment of Pain in Patients Suspected Endometriosis	Wrong publication type
	The effect of garlic tablets on the pain of women with endometriosis	Wrong publication type
	The effect of vitamin D supplementation in treatment of endometriosis	Wrong publication type
Liang 2014	Influence on dysmenorrhea of Bushen Tiaojing Huoxue decoction combined with enema in treatment of endometriosis	Could not access
	A multicentre, randomised, double-blind, placebo-controlled clinical trial to evaluate the use of Chinese herbal medicine in the management of endometriosis: a prospective study	Wrong publication type

Mardon AK, Leake HB, Hayles C, Henry M, Neumann T, Moseley GL, Chalmers KJ
Corresponding author: k.jane.chalmers@gmail.com

The efficacy of self-management strategies for females with endometriosis: a systematic review
Reproductive Sciences

Supplementary file 2: Excluded full-text articles

Yin 2016	Treating 49 cases of endometriosis with laparoscopy in combination with chinese medicine	Could not access
Behboodi Moghadam 2016	Effect of garlic on dysmenorrhea in women with endometriosis	Could not access
Luo 1996	A clinical study of Luo's Nei Yi Fang for Endometriosis	Could not access
	Multi-center Clinical Trials of Sanjie Analgesic Capsule in Treating Endometriosis	Wrong publication type
	The effect of isoflavones in endometriotic pain	Wrong publication type
Behboodi Moghadam 2016	Effect of garlic on dysmenorrhea in women with endometriosis	Wrong publication type
	A Clinical Trial to Test a Modified Traditional Chinese Herbal Medicine for the Treatment of Endometriosis	Wrong publication type
Hernandez 2012	Effects of Diet, Exercise, and Weight on Endometriosis Pain Reduction	Wrong publication type
Ferrero 2010	Norethisterone acetate in the treatment of colorectal endometriosis: A pilot study	Wrong intervention
Sesti 2009	Postoperative dietary supplementation with omega-3 fatty acids and antioxidants after conservative surgery for symptomatic endometriosis	Wrong publication type
Arndt 2007	Alternative therapy options for endometriosis	Not English
Santanam 2013	Antioxidant supplementation reduces endometriosis-related pelvic pain in humans	Wrong patient population
Santanam 2013	Antioxidant supplementation reduces endometriosis-related pelvic pain in humans	Wrong patient population
Lo Monte 2013	[Administration of MICRONIZED PALMITOYLETHANOLAMIDE (PEA)-transpolydatin in the treatment of chronic pelvic pain in women affected by endometriosis: preliminary results.]	Not English
Ernst 2013	Chinese herbs against endometriosis?	Not English
Kuhlmann 2019	Chinese Medicine for Endometriosis, Dysmenorrhea and Fibroids	Not English
Kavtaradze 2003	Vitamin E and C supplementation reduces endometriosis related pelvic pain	Wrong publication type
Antoniadis 2020	Influence of mindfulness-based Stress Reduction on Patients with Endometriosis in a randomized Cohort Study	Not English
Borghini 2020	Irritable Bowel Syndrome-Like Disorders in Endometriosis: Prevalence of Nickel Sensitivity and Effects of a Low-Nickel Diet. An Open-Label Pilot Study	Wrong patient population
Flower 2011	A feasibility study exploring the role of Chinese herbal medicine in the treatment of endometriosis	Wrong intervention
Fisher 2018	Cyclic Perimenstrual Pain and Discomfort and Australian Women's Associated Use of Complementary and Alternative Medicine: A Longitudinal Study	Wrong patient population
Sesti 2011	Nutritional regimen and antioxidants in the treatment of symptomatic pelvic endometriosis	Wrong publication type
	Endometriosis in Patients With Irritable Bowel Syndrome: Specific Symptomatic and Demographic Profile, and Response to the Low FODMAP Diet	Wrong patient population

Mardon AK, Leake HB, Hayles C, Henry M, Neumann T, Moseley GL, Chalmers KJ
Corresponding author: k.jane.chalmers@gmail.com

The efficacy of self-management strategies for females with endometriosis: a systematic review
Reproductive Sciences

Supplementary file 2: Excluded full-text articles

Mills 2011	A conundrum: Wheat and gluten avoidance and its implication with endometriosis patients	Wrong publication type
Indraccolo 2010	Effect of palmitoylethanolamide-polydatin combination on chronic pelvic pain associated with endometriosis: Preliminary observations	Wrong patient population
	A randomized controlled trial for Yi-Sheng Xiao-Zheng granules in the treatment of dysmenorrhea caused by pelvic endometriosis (syndrome of kidney deficiency and blood stasis)	Could not access
Weng 2015	Chinese medicinal plants for advanced endometriosis after conservative surgery: a prospective, multi-center and controlled trial	Wrong outcome measures
Lorenz 2016	Transcutaneous electrical nerve stimulation in deep infiltrating endometriosis	Not English
Cobellis 2004	The treatment with a COX-2 specific inhibitor is effective in the management of pain related to endometriosis	Wrong intervention
Zhang 2017	Jing Tong Yu Shu, a traditional Chinese medicine, suppresses IL-1β and IL-6 gene expressions in macrophages, and alleviates endometriosis	Wrong study design
Mesrine 2013	Re: "Dairy-food, calcium, magnesium, and vitamin D intake and endometriosis: a prospective cohort study"	Wrong publication type
Hansen 2015	Long-time effects of mindfulness-based psychological treatment on chronic pain and quality of life in endometriosis	Wrong publication type
Yang 2006	Comparative study on the efficacy of Yiweining and Gestrinone for post-operational treatment of stage III endometriosis	Wrong intervention
Baek 2020	Vitamin D and endometriosis: Is there a correlation with disease severity?	Wrong publication type
Shoebotham 2016	Therapeutic affordances of online support group use in women with endometriosis	Wrong patient population
Nodler 2020	Supplementation with vitamin D or omega-3 fatty acids in adolescent girls and young women with endometriosis (SAGE): a double-blind, randomized, placebo-controlled trial	Other
Kiesel 1989	Treatment of endometriosis	Not English
Giugliano 2013	The adjuvant use of N-palmitoylethanolamine and transpolydatin in the treatment of endometriotic pain	Wrong patient population
Korell 2011	Endometriosis and Diet	Not English
Kold 2012	Mindfulness-based psychological intervention for coping with pain in endometriosis	Wrong intervention
Kold 2016	Mindfulness-based psychological intervention for coping with pain in endometriosis (vol 64, pg 2, 2012)	Wrong intervention
Lo Monte 2013	[Administration of micronized palmitoylethanolamide (PEA)-transpolydatin in the treatment of chronic pelvic pain in women affected by endometriosis: preliminary results]	Not English

Mardon AK, Leake HB, Hayles C, Henry M, Neumann T, Moseley GL, Chalmers KJ
Corresponding author: k.jane.chalmers@gmail.com

The efficacy of self-management strategies for females with endometriosis: a systematic review
Reproductive Sciences

Supplementary file 2: Excluded full-text articles

Lete 2018	Effectiveness of an antioxidant preparation with N-acetyl cysteine, alpha lipoic acid and bromelain in the treatment of endometriosis-associated pelvic pain: LEAP study	Wrong patient population
	Corrigendum Mindfulness-based psychological intervention for coping with pain in endometriosis (Nordic Psychology, 64, (2012), (2-16), DOI:10.1080/19012276.2012.693727)	Wrong publication type
Jankovich 2017	The low FODMAP diet reduced symptoms in a patient with endometriosis and IBS	Wrong outcome measures
Hawkins 2003	The Use of Thermal Biofeedback in the Treatment of Pain Associated with Endometriosis: Preliminary Findings	Wrong patient population
Zhao 2013	Chinese medicine improves postoperative quality of life in endometriosis patients: a randomized controlled trial	Wrong intervention
Swarnakar 2007	chroniCurcumin arrests endometriosis by downregulation of matrix metalloproteinase-9 activity	Wrong study design
Mehdizadehkashi 2021	The effect of vitamin D supplementation on clinical symptoms and metabolic profiles in patients with endometriosis	Wrong patient population
	Mindfulness Meditation for Chronic Pelvic Pain Management	Wrong patient population
Awad 2017	Efficacy of exercise on pelvic pain and posture associated with endometriosis: within subject design	Wrong intervention
Carpenter 1995	The effect of regular exercise on women receiving danazol for treatment of endometriosis	Wrong outcome measures

Mardon AK, Leake HB, Hayles C, Henry M, Neumann T, Moseley GL, Chalmers KJ
Corresponding author: k.jane.chalmers@gmail.com
