# Supplementary material for: The Efficacy of Self-Management Strategies for Females with Endometriosis: a Systematic Review
Source: Reprod Sci. 2022 Apr 29;30(2):390–407. doi: 10.1007/s43032-022-00952-9 (PMC9988721; doi:10.1007/s43032-022-00952-9)
Supplement: Supplementary file 3 — Supplementary file3 (PDF 73 KB) [file 43032_2022_952_MOESM3_ESM.pdf]

Supplementary file 3: Study raw data

Study	Comparison	RoB	Dysmenorrhoea			Non-menstrual pelvic pain			Dyspareunia			Overall pain		
			Intervention	Control		Intervention	Control		Intervention	Control		Intervention	Control	
			mean (SD); n	mean (SD); n	SMD [95% CI]	mean (SD); n	mean (SD); n	SMD [95% CI]	mean (SD); n	mean (SD); n	SMD [95% CI]	mean (SD); n	mean (SD); n	SMD [95% CI]
**Dietary supplements**														
Almassinokiani 2016	Vitamin D vs Placebo (End of intervention)		2.1 (2.33); 19	2.73 (2.84); 19	-0.24 [-0.88, 0.40]	0.84 (1.74); 19	0.68 (1.7); 19	0.09 [-0.55, 0.73]						
Khodaverdi 2019	Lactobacillus vs Placebo (End of intervention)		3.07 (2.34); 16	4.47 (2.13); 16	-0.58 [-1.29, 0.13]	2 (1.93); 16	2.5 (2.34); 16	-0.23 [-0.92, 0.47]	2.55 (2.77); 16	3.25 (2.3); 16	-0.27 [-0.96, 0.43]			
	Lactobacillus vs Placebo (Short term)		3.8 (2.54); 16	4.6 (1.92); 16	-0.35 [-1.05, 0.35]	3 (2.39); 16	2.44 (2.13); 16	0.24 [-0.45, 0.94]	3.09 (2.54); 16	3.17 (2.08); 16	-0.03 [-0.73, 0.66]			
Kohama 2007	Pycnogenol vs GnRHa (End of intervention)		2.2 (0.59); 24	2.3 (0.66); 20	-0.16 [-0.75, 0.44]	2.1 (0.63); 23	2.5 (0.89); 20	-0.52 [-1.13, 0.09]						
Maia 2012	Resveratrol vs OC (End of intervention)		0.2 (0.4); 12	2.1 (0.5); 12	-4.05 [-5.54, -2.56]									
Nodler 2020	Vitamin D vs Placebo (End of intervention)											5.5 (3.2); 23	4.4 (3.1); 19	0.34 [-0.27, 0.95]
	Fish oil vs Placebo (End of intervention)											5.2 (3.3); 17		0.24 [-0.41, 0.90]
Schwertner 2013	Melatonin vs Placebo (End of intervention)		4.24 (2.61); 20	6.84 (2.38); 20	-1.02 [-1.68, -0.36]				4.68 (2.61); 20	6.08 (1.42); 20	-0.65 [-1.29, -0.02]	2.78 (1.35), 20	4.58 (4.58); 20	-1.25 [-1.94, -0.57]
**Dietary modifications**														
Sesti 2007	Diet therapy vs Placebo (Intermediate)			6.4 (1.3); 110	0 [-0.38, 0.38]		6.2 (0.9); 110	-1.57 [-1.99, -1.15]		4.8 (1.2); 110	0.17 [-0.21, 0.55]			
	Diet therapy vs Gn-RHa (Intermediate)		6.4 (1); 35	5.9 (0.9); 39	0.52 [0.06, 0.99]	4.7 (1.1); 35	5 (1.1); 39	-0.27 [-0.73, 0.19]	5 (1.1); 35	4.3 (1.2); 39	0.6 [0.13, 1.07]			
	Diet therapy vs OC (Intermediate)			5.5 (1.2); 38	0.8 [0.32, 1.28]		5 (0.8); 38	-0.31 [-0.77, 0.15]		4.5 (1.3); 38	0.52 [0.05, 0.98]			
**Exercise**														
Goncalves 2016	Yoga vs No yoga (End of intervention)											3.5 (3); 15	6.5 (2.9) 8	-0.97 [-1.89, -0.06]

Effect size calculations

CI, confidence interval; GnRHa, gonadotropin-releasing hormone agonist; OC, oral contraceptive; RoB, risk of bias; SD, standard deviation; SMD, standardised mean difference
